# Biomass Allocation in *Gentianella turkestanorum* is Driven by Environmental Factors and Functional Traits

**DOI:** 10.3390/plants13243463

**Published:** 2024-12-11

**Authors:** Qingzhen Sun, Enzhao Wang, Xiaoling Fan, Bin Liu

**Affiliations:** 1 College of Life Sciences, Xinjiang Normal University, Urumqi 830017, China; sunqz2535@163.com (Q.S.); wez210127@163.com (E.W.); fxl0518@126.com (X.F.); 2 Xinjiang Key Laboratory of Special Species Conservation and Regulatory Biology, Urumqi 830017, China; 3 Key Laboratory of Special Environment Biodiversity Application and Regulation in Xinjiang, Urumqi 830017, China

**Keywords:** biomass allocation, elevation, soil factors, functional traits, *Gentianella turkestanorum*

## Abstract

Exploring the elevation distribution characteristics, biomass allocation strategies, and the effects of elevation, soil factors, and functional traits on the biomass of *Gentianella turkestanorum* (Gand.) Holub is of great significance for the production, development, utilization, and protection of the medicinal material resources. In this study, we investigated the biomass and functional traits of the root, stem, leaf, and flower of *G. turkestanorum*, analyzing their elevation distribution patterns, allometric growth trajectories, and their correlations. The results showed that the biomass of different organs of *G. turkestanorum* decreases with increasing elevation, and the belowground biomass/aboveground biomass increases with elevation. The flower biomass accounts for 59.24% of the total biomass, which was significantly higher than that of other organs. *G. turkestanorum* biomass follows the optimal allocation theory, adopting a ‘pioneering’ growth strategy at low elevations and a ‘conservative’ strategy at high elevations. Chlorophyll content and leaf thickness of *G. turkestanorum* were positively correlated with elevation, but leaf dry matter content and the number of flowers were negatively correlated with elevation. Compared to functional traits, elevation and soil factors have a stronger explanatory power regarding the biomass of *G. turkestanorum*. Elevation, soil moisture content, pH, available phosphorus, total nitrogen, and ammonium nitrogen significantly affect the biomass of *G. turkestanorum*, with only pH showing a positive correlation with biomass. Among these factors, elevation, soil moisture content, and pH significantly impact the accurate prediction of *G. turkestanorum* biomass. The number of flowers, crown width, root length, root diameter, and leaf dry matter content all have a significantly positive correlation with the biomass of *G. turkestanorum*, with the number of flowers and root diameter making significant contributions to the accurate prediction of biomass. Elevation can directly affect the biomass of *G. turkestanorum* and can also indirectly affect it through other pathways, with the direct effect being greater than the indirect effect.

## 1. Introduction

*Gentianella turkestanorum* (Gand.) Holub, a member of the family Gentianaceae, is an annual or biennial herbaceous plant that is extensively used in traditional Chinese medicine. It is renowned for its therapeutic properties, which include clearing heat toxins, detoxification, reducing dampness, and alleviating swelling. The whole plant is used to make tea to treat symptoms such as cold and fever [1]. Modern medicine has proved that *G. turkestanorum* contains chemical components that can reduce blood sugar, improve the immune system [2], and has antibacterial and anti-inflammatory effects [3]. Although *G. turkestanorum* possesses a high medicinal value, its harvesting, cultivation, and production processes lack standardized management. Concurrently, the severe degradation of grasslands attributed to global warming has led to a significant decline in the herb resources of *G. turkestanorum*. The current research focuses on the extraction process of iridoid glycosides [4], evaluation methods, determination of chemical composition content [1], and pharmacological mechanism [3]. Regarding the biological characteristics of *G. turkestanorum*, especially the factors influencing biomass, there have been few reports.

The uneven distribution of plant resources within the internal structure and functional components reflects changes in plant growth and metabolism, and is also an important reflection of plant response to environmental changes [5]. Meanwhile, the allocation strategy of biomass among plant organs has also attracted extensive attention from many scholars [6,7,8]. Currently, there are two main hypotheses regarding plant biomass allocation. The optimal allocation theory posits that plants can respond to environmental changes by adjusting the relative allocation of each organ, thereby allocating more biomass to the organ that can best capture limited resources 8. In contrast, the allometric distribution theory suggests that the distribution of plant biomass is solely a function of plant size and is determined by a power function that is independent of environmental changes [9].

Elevation greatly affects the biomass allocation strategy. A study on the biomass allocation strategy of *Gentiana rigescens* on the Yunnan–Guizhou Plateau found that plants growing at high elevations allocate proportionately more biomass to reproduction at larger sizes and less at smaller sizes than plants growing at lower elevations [10]. The sexual reproduction distribution of *Gentiana hexaphylla* was positively correlated with elevation [11]. Soil is the main source of nutrients obtained by plants, and soil moisture content, pH, nitrogen, phosphorus and potassium, organic matter content, and microbial activity all affect the production of plant biomass [12,13]. Plant functional traits can be used as indicators of the ecological function of species, which can be used to understand the resource utilization strategies and environmental adaptability of species, and can also be used to study the effects of abiotic stress on ecosystem functions and services [14]. Bynum and Smith [15] found that the flower diameter of *Gentiana algida* in a high elevation population was larger than that in a low elevation population, and the flower diameter of *Gentiana hexaphylla* in the Tibetan Plateau increased with the increase in elevation. The opposite is true for plant height [11]. A study on riparian area biomass affected by flooding stress due to dam construction found that aboveground biomass was positively correlated with leaf dry matter content, negatively correlated with specific leaf area, and had no significant correlation with root traits [16]. Plant height, leaf area, specific leaf area, and leaf weight had a direct positive effect on the total biomass of *Bistorta macrophylla* in alpine grassland [17]. Therefore, in view of the medicinal and ecological value of *G. turkestanorum* [18], it is necessary to carry out research on its biomass allocation strategy and influencing factors, which can not only enrich the understanding of the growth strategy of *G. turkestanorum* in elevation gradient, but also provide theoretical basis for the protection, management, and sustainable production and utilization of *G. turkestanorum*. Furthermore, it provides a broader theoretical basis for the effects of environmental factors and functional traits on grassland medicinal plants.

**Figure 1:**
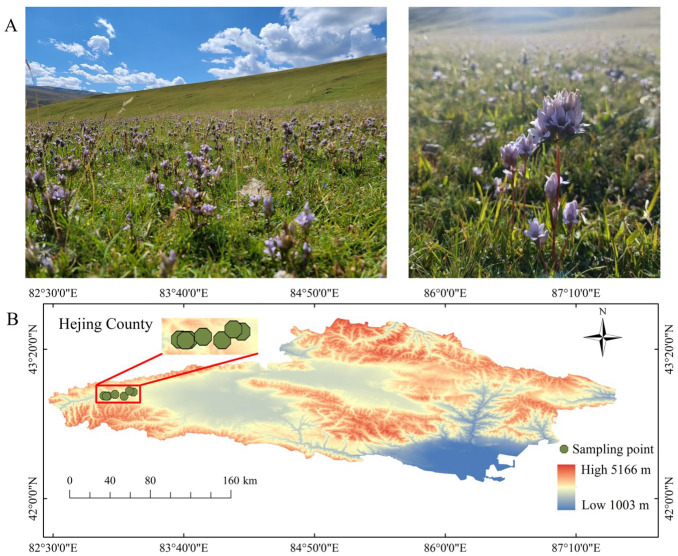
Habitat photo (**A**) and sampling sites (**B**) of *Gentianella turkestanorum* (Gand.) Holub.

Located in the northwest of Xinjiang, Bayanbulak grassland is the second largest grassland in China, a key gene pool for species in the region. It is a typical meadow grassland with flat terrain, elevation difference, and rich species resources, among which *G. turkestanorum* is widely distributed and abundant 18 (Figure 1A), making Bayanbulak grassland an ideal place to study the effects of elevation, soil, and functional traits on the biomass of *G. turkestanorum*. Therefore, in this study, the aim is to explore the following questions: (1) what is the elevation pattern of different organ biomass and allocation strategies of *G. turkestanorum*? (2) how do the functional traits of different organs of *G. turkestanorum* change with elevation? (3) how do elevation, soil factors, and functional traits affect the biomass of *G. turkestanorum*?

## 2. Materials and Methods

### 2.1. Study Area and Sites

Bayanbulak grassland (83°42′ E–85°51′ E, 42°59′ N–43°07′ N) is located in the northwestern Bayangguo Mongolian Autonomous Prefecture HeJing County, Xinjiang Uygur Autonomous Region. It is 136 km wide from north to south and 270 km long from east to west, with a total area of 2.3 × 10^4^ km^2^. Located on the southern slope of Tianshan Mountain, the regional elevation is 2000–3600 m, with a certain elevation difference. The annual precipitation of Bayanbulak grassland is 276.2 mm, while the annual evaporation ranges from 1022.9 mm to 1247.5 mm. The average annual temperature is −4.7 °C, the average annual wind speed is 2.6 ms^−1^, and the sunshine duration is 2466–2616 h [19]. Bayanbulak Grassland, encircled by snow-capped mountains, primarily relies on snow and ice meltwater, as well as rainfall, for its water supply. The main soil types were alpine meadow soil and meadow steppe soil, and the vegetation species taxa in the study area were mainly *Gentianaceae*, *Gramineae*, *Cyperaceae*, and *Polygonaceae, G. turkestanorum* occupied an important ecological niche [18].

### 2.2. Field Investigation, Sample Setting, and Sample Collection

We conducted a survey of *G. turkestanorum* in August 2023, which was the flowering period of *G. turkestanorum*. The sample plots were set along the elevation gradient, approximately 100 m apart, based on the suitability of *G. turkestanorum*. A total of seven elevation sample sites were set up at 2405, 2534, 2635, 2688, 2805, 2906, and 3000 m (Figure 1B). Each sample plot contained three large squares, each measuring 10 × 10 m, with approximately 100 m horizontal intervals between the large squares. The small squares were set according to the plum-shaped five-point sampling method, and the small squares were set in the large squares according to the specifications of 1 × 1 m. A total of 105 small squares were set. The population density (number per unit area) of *G. turkestanorum* in the small quadrat was recorded, plant height (H) and crown diameter (CD) were measured with a tape measure, and all *G. turkestanorum* in the small quadrat were collected by the whole-plant harvesting method. Attention was paid to protecting the integrity of the roots when digging, and the samples were then put into collection bags and numbered. After removing soil surface impurities, the 7.5 cm ring knife method was used to dig the soil 0–30 cm deep into the soil in the large sample square according to the diagonal principle. The soil samples were then placed into collection bags and numbered. A total of 63 soil samples were collected.

### 2.3. Sample Treatment of Plant and Soil

The plant samples were classified according to different elevations and different small quadrates. The individual plants of *G. turkestanorum* were divided into the four parts of root, stem, leaf, and flower in the laboratory, each part was then numbered and recorded, and subsequently dried in the oven at 65 °C to a constant weight. The dry weight represented the biomass per unit area of different organs of *G. turkestanorum*, namely root biomass (RB), stem biomass (SB), leaf biomass (LB), and flower biomass (FB). TB (total biomass) is the sum of the biomass of these four organs. BGB (belowground biomass) is the root biomass, and AGB (aboveground biomass) is the sum of SB, LB, and FB.

The soil samples were taken back to the laboratory for air-drying at room temperature, and impurities in the air-dried soil were removed. After grinding by a ball mill (Retsch MM400, Retsch GmbH, 42781 Haan, Germany), the samples were sifted (0.15 mm) and put into a sealed bag for the subsequent determination of soil physical and chemical indexes.

### 2.4. Determination of Functional Traits

Five mature and evenly sized *G. turkestanorum* were randomly selected as sample plants in each small square (1 × 1 m) for the determination of functional traits. Root length (RL), root diameter (RD), stem length (SL), and stem diameter (SD) were measured with a ruler (accuracy 0.1 cm) and Vernier caliper (accuracy 0.01 mm). According to the field investigation, most of the *G. turkestanorum* had an obvious main root and main stem, and fewer fibrous roots and lateral stems. Therefore, the average measurement results of RL, SL as the main root and main stem length, RD and SD as the main root and the upper, middle, and lower parts of the main stem were obtained. Three healthy, fully extended and evenly sized leaves were selected on each sample plant from the base and middle of the stem; the chlorophyll content (CHL) of the leaf was measured by SPAD-502 portable chlorophyll analyzer (Konica Minolta, Langenhagen, Germany); leaf thickness (LT) was measured by Vernier caliper. Three points were taken for each sample leaf, avoiding leaf veins, and the average value was taken. Twenty healthy, complete and evenly sized leaves were randomly selected from the large sample and put into storage bags with a small amount of deionized water and brought back to the laboratory. The fresh weight of the leaf was measured by an electronic scale with an accuracy of 0.0001 g, and the average was the fresh weight of a single leaf. Then, three leaves were randomly selected and then scanned using a scanner (UNIS K3000C; Unisplendour Corporation Limited, Beijing, China) and the blade area was calculated using Image-J software Version 1.8.0. The mean value was determined to be the leaf area (LA). Finally, the 20 collected leaves were dried to a constant weight at 65 °C, and the mean value was determined to be the dry weight of single leaf. Specific leaf area (SLA) = leaf area/leaf dry weight; leaf dry matter content (LDMC) = (leaf dry weight/leaf fresh weight) × 100%. The average number of flowers was the number of flowers per plant, number of flowers (NOFs) = number of flowers per plant × population density, that is, the number of flowers per unit area of *G. turkestanorum*. Nine flowers were randomly selected from the sample plants and their crown diameter was measured by a ruler. The mean was the crown diameter of flowers (COFs).

### 2.5. Determination of Soil Physical and Chemical Indexes

Soil samples after grinding were tested for soil physical and chemical properties [20,21], soil total carbon content (TC) was determined by the potassium dichromate volumetric external heating method. Soil total nitrogen content (TN) was determined by HClO_4_-H_2_SO_4_ digestion method and the FOSS 1035 automatic Kjeldahl nitrogen analyzer (KDN-102F; FOSS, Shanghai, China). Soil ammonium nitrogen content (AN) was determined by 2 mol·L^−1^ KCl extraction and indophenol blue colorimetry. Soil nitrate nitrogen content (NN) was determined by dual-wavelength ultraviolet spectrophotometry. Soil total phosphorus content (TP) was determined by acid-soluble-molybdenum-antimony reactance colorimetric method using Agilent CARY60 ultraviolet spectrophotometer (Agilent, Santa Clara, CA, USA). Soil-available phosphorus content (AP) was determined by NaOH extraction and molybdenum-antimony resistance colorimetric method with Agilent CARY60 ultraviolet spectrophotometer. Soil total potassium content (TK) was dissolved by NaOH and determined by flame spectrophotometry. Soil-available potassium content (AK) was determined by flame atomic absorption spectrometry. The soil pH value was determined by a pH meter (PHSJ-6L; Shanghai Yidian Technology, Shanghai, China), and the soil water content (SW) was obtained by drying weighing method.

### 2.6. Data Processing and Analysis

The normal distribution of the data was tested using the “mice” software package in R 4.3.0, and data that did not conform to a normal distribution underwent logarithmic transformation. The “agricolae” software package was utilized to assess the significance of variations in biomass and functional traits across different elevations for the organs. The “corrplot” software package was used to analyze the correlation among functional traits.

The elevation was divided into the following three elevation gradients: low (2400–2500 m), middle (2600–2700 m), and high (>2800 m). Standardized major axis regression (SMA) was used for regression analysis of the allometry equation of biomass allocation among organs [9], the allometric equation is expressed as follows: log y = a × log x + b, where log x is the logarithm of the biomass of one organ, log y is the logarithm of the biomass of another organ, a is the allometric slope, and b is the allometric intercept. The “smatr” software package was used to test differences between allometric equations. If the allometric growth trajectory is unchanged, biomass allocation is only affected by plant size, which follows the allometric allocation theory. When the allometric growth trajectory changes, the biomass allocation is affected by environmental changes and follows the optimal allocation theory.

Since we have considered many influencing factors for the biomass of *G. turkestanorum*, and there is a significant correlation among these factors, which may lead to multicollinearity, we used “tidyverse” and “caret” software packages to calculate variance inflation factor (VIF). Soil and functional trait factors with VIF ≥ 10 were removed to reduce their multicollinearity. Variance partition analysis (VPA) was conducted using the “vegan” software package to identify the main influencing factors driving the biomass of *G. turkestanorum*. Redundancy analysis (RDA) was performed using vegan and “ecodist” software packages to investigate the correlation of elevation, soil factors, functional traits, and biomass of *G. turkestanorum*. rfPermute and A3 software packages were used to construct a random forest model, and then the contributions of allocation-soil factors and functional diversity to the biomass prediction accuracy of *G. turkestanorum* were analyzed. Factors significantly impacting biomass were identified as key variables for constructing the piecewiseSEM model based on RDA results, and the model was constructed using the software packages “nlme”, “lme4”, “piecewiseSEM” and “QuantPsyc”. Furthermore, the direct and indirect effects of elevation, soil factors, functional traits, and biomass were explored, and the marginal and conditional contributions of the predictors in driving the biomass of *G. turkestanorum* were provided. Fisher’s C test (when 0.05 < *p* < 1.00) was used to confirm the goodness of the modeling results, and the stepwise regression method, the significance of the model (*p* < 0.05), and the goodness of the model were combined to adjust and modify the model [22].

## 3. Results

### 3.1. Variations in Organ Biomass and BGB/AGB Ratio Along the Elevation Gradient

**Figure 2:**
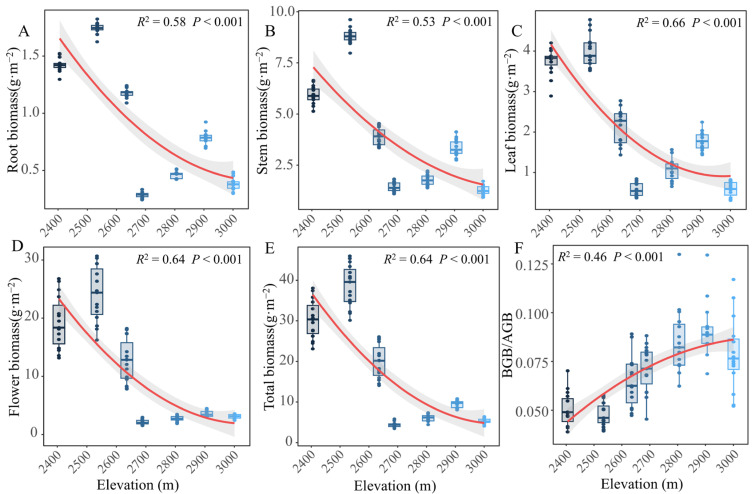
Elevation distribution characteristics of biomass and BGB/AGB in different organs. The red line indicates a linear fit. BGB/AGB: belowground biomass/aboveground biomass. Elevation distribution characteristics of root (**A**), stem (**B**), leaf (**C**), flower (**D**), total biomass (**E**) and BGB/AGB (**F**).

The results of the one-way ANOVA showed that the BGB/AGB showed a gradually increasing trend with the increase in elevation (Figure 2F, Table S1). On the whole, the root, stem, leaf, flower, and total biomass of *G. turkestanorum* increased first then decreased with the increase in elevation, and reached the peak value at 2534 m. Furthermore, according to the linear fitting results, the biomass of different organs of *G. turkestanorum* showed a gradual decline with the increase in elevation (Figure 2). It can be concluded that compared with the high elevation, a low elevation is more suitable for the growth of *G. turkestanorum*.

### 3.2. Allometric Growth Trajectory of Organ Biomass Along the Elevation Gradient

**Figure 3:**
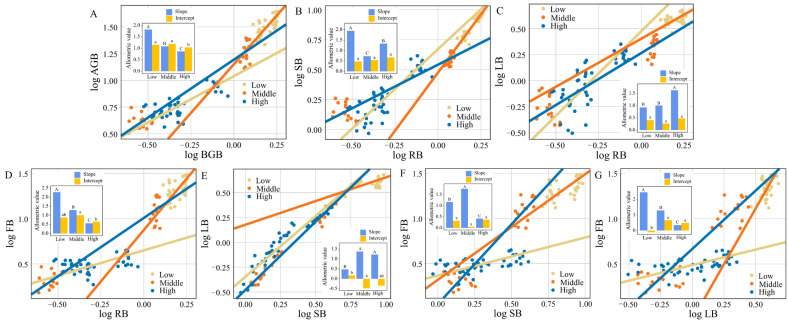
Allometric growth trajectories of different organ biomass of *G. turkestanorum* at different elevation gradients. (**A**) log AGB–log BGB; (**B**) log SB–log RB; (**C**) log LB–log RB; (**D**) log FB–log RB; (**E**) log LB–log SB; (**F**) log FB–log SB; (**G**) log FB–log LB. In the histograms, uppercase letters denote differences in slopes and lowercase letters denote differences in intercepts among different elevations gradients.

SMA results (Figure 3, Table 1) showed significant changes in the allometric growth trajectories of the biomass of various organs of *G. turkestanorum*. In the relationship between BGB and AGB (Figure 3A, Table 1), the slope decreased significantly as elevation increased (*p* < 0.001), and the intercept of high elevation was significantly lower than that of middle and low elevation (*p* < 0.001), indicating that with the increase in elevation, *G. turkestanorum* gradually reduced the allocation of the aboveground biomass, and gradually increased the allocation of the belowground biomass.

### 3.3. Variations in Functional Traits Along the Elevation Gradient

**Figure 4:**
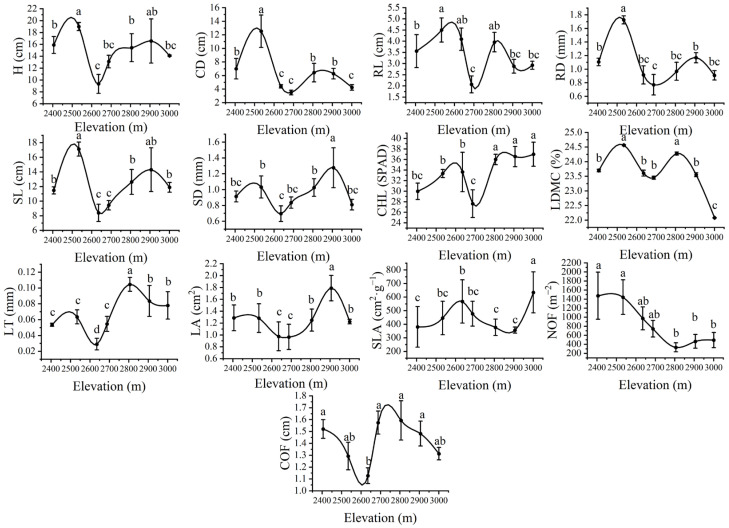
Elevation distribution of functional traits. H: plant height; CD: crown diameter; RL: root length; RD: root diameter; SL: stem length; SD: stem diameter; CHL: chlorophyll; LDMC: leaf dry matter content; LT: leaf thickness; LA: leaf area; SLA: specific leaf area; NOFs: number of flowers: COFs: crown diameter of flowers. The same below.

The results of one-way ANOVA on the functional traits of *G. turkestanorum* showed that plant height, crown width, root length, root diameter, stem length, and leaf dry matter content showed a bimodal trend with the increase in elevation, and the peak value at the low elevation of 2534 m was significantly higher than at high elevation sites (*p* < 0.05). Stem diameter, chlorophyll content, leaf thickness, and leaf area also showed a bimodal trend with the increase in elevation, but the peak value at high elevation sites was significantly higher than at low elevation sites (*p* < 0.05). The specific leaf area increases gradually with the increase in elevation, and reaches the highest value at an elevation of 3000 m. The flower number, decreasing with the increase in elevation, was highest at an elevation of 2405 m. The corolla width decreased at first and then increased, but was lowest at 2635 m elevation (Figure 4, Table S2).

**Figure 5:**
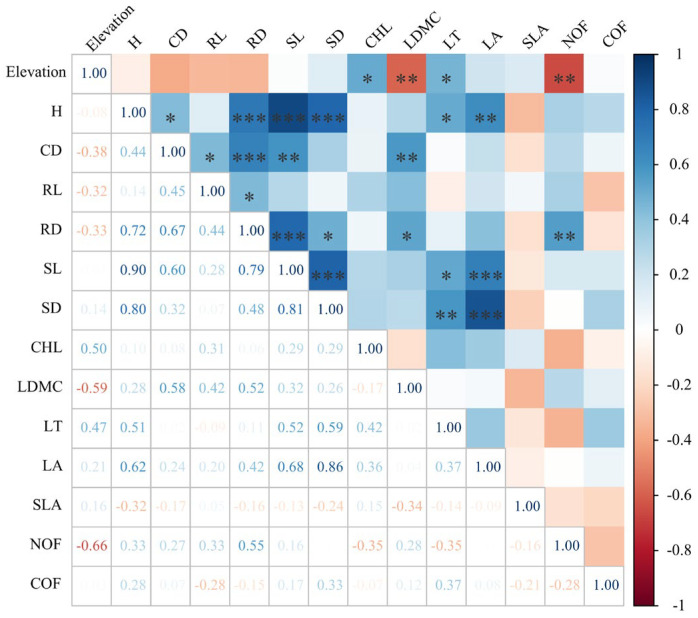
Pearson correlation between functional traits and elevation. Blue indicates positive correlation, red indicates negative correlation, *: *p* < 0.05; **: *p* < 0.01; ***: *p* < 0.001. H: plant height; CD: crown diameter; RL: root length; RD: root diameter; SL: stem length; SD: stem diameter; CHL: chlorophyll; LDMC: leaf dry matter content; LT: leaf thickness; LA: leaf area; SLA: specific leaf area; NOFs: number of flowers; COFs; crown diameter of flowers.

Pearson correlation analysis showed that the chlorophyll content and leaf thickness were positively correlated with elevation (*p* < 0.05), but leaf dry matter content and flower number were negatively correlated with elevation (*p* < 0.01) (Figure 5).

### 3.4. Elevation-Soil Factors, Functional Traits and Biomass Relationships

**Figure 6:**
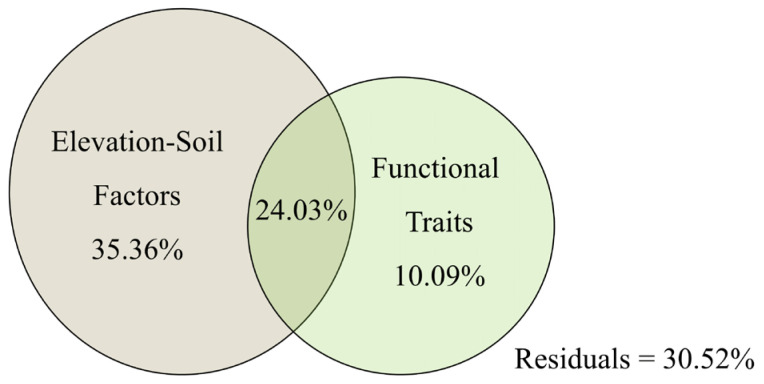
Variance decomposition results of biomass of *G. turkestanorum*.

Variance decomposition revealed elevation, soil factors, and functional traits were the main factors driving the biomass of *G. turkestanorum*, with a total explanatory value of 69.84%. The elevation and soil factors were the primary drivers of biomass change, accounting for 59.39% of the biomass change, the functional traits independently explained 10.09% of the biomass change, and the interaction of elevation and soil factors explained 24.03% of the biomass change in *G. turkestanorum* (Figure 6).

**Figure 7:**
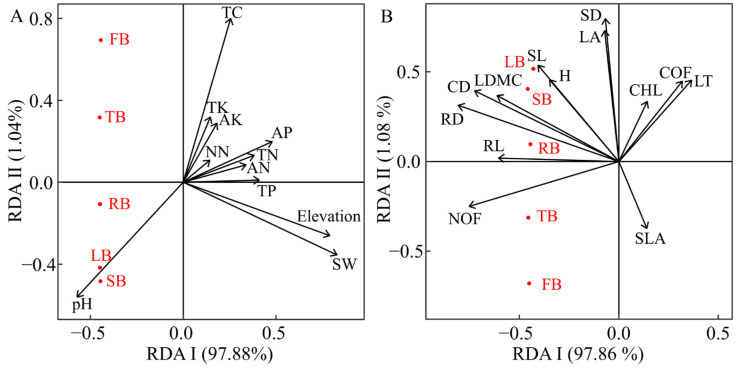
RDA ranking of biomass and elevation-soil factors (**A**) and functional traits (**B**) of *G. turkestanorum*. TC: soil total carbon content; TN: soil total nitrogen content; TP: soil total phosphorus content; TK: soil total potassium content; AN: soil ammonium nitrogen content; NN: soil nitrate nitrogen content; AP: soil available phosphorus content; AK: soil available potassium content; SW: soil water content; pH: soil ph.

**Figure 8:**
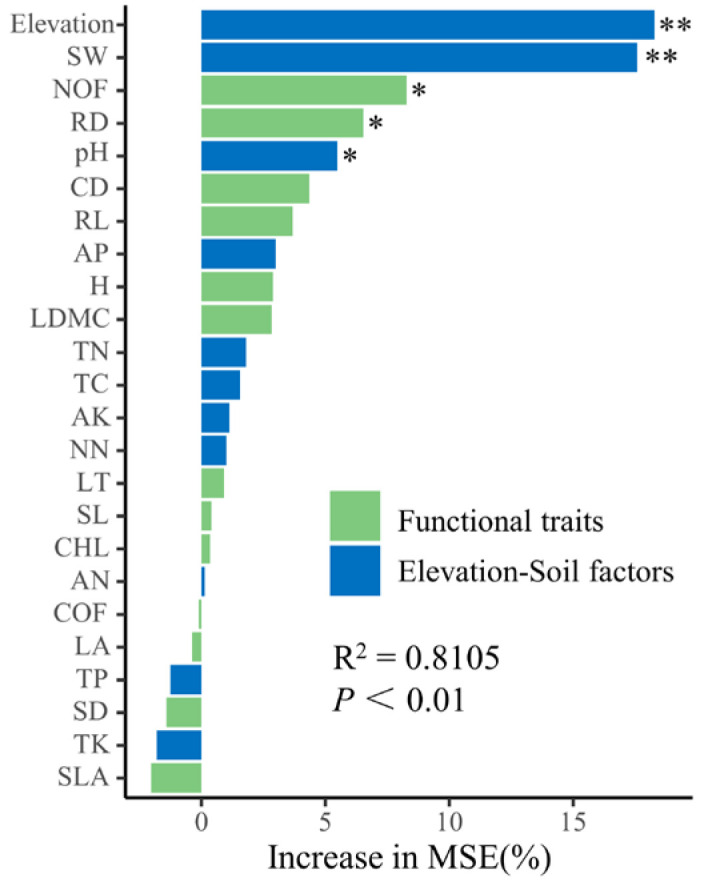
Analysis of biomass and elevation-soil factors and functional traits by random forest model in *G. turkestanorum*. “Increase in MSE” means that when a factor is removed, the accuracy of predicting biomass decreases. *: *p* < 0.05; **: *p* < 0.01.

Redundancy analysis and random forest modeling revealed that among the elevation and soil factors affecting the biomass of *G. turkestanorum*, elevation, soil moisture content, pH, available phosphorus, total nitrogen, and ammonium nitrogen had significant effects on the biomass of *G. turkestanorum* in the study area; among these factors, only the pH (7.15–7.70) was significantly positively correlated with the biomass (Figure 7A, Table 2 and Table S3). The elevation, soil moisture content, and pH had significant effects on the accurate prediction of the biomass of *G. turkestanorum* (Figure 8). Among the functional traits, flower number, crown width, root length, root thickness, and leaf dry matter content had a significant positive correlation with the biomass of *G. turkestanorum* in the study area, but it had no significant correlation with other functional traits (Figure 7B, Table 2). Flower number and root diameter contributed significantly to the accurate prediction of the biomass of *G. turkestanorum* (Figure 8).

**Figure 9:**
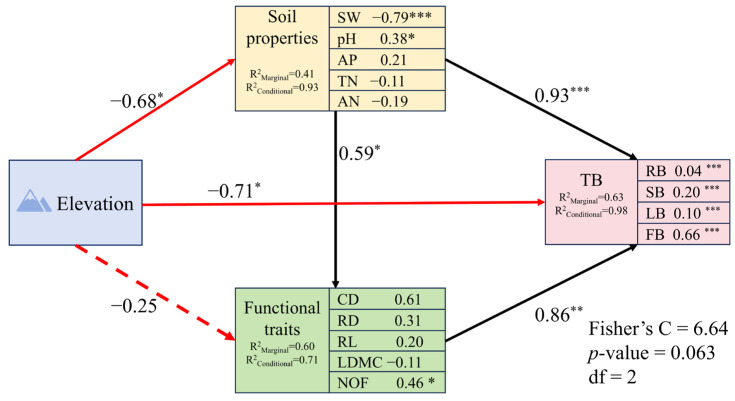
Direct and indirect drivers of biomass of *G. turkestanorum*. The number next to the arrow is the normalized path coefficient, which represents the size of the direct normalized effect of the relationship (positive values represent positive effects and negative values represent negative effects). The black lines represent positive effects and the red lines represent negative effects. The solid line means there is a significant correlation, and the dashed line means there is no significant correlation. The respective significance of each predictor was * *p* < 0.05, ** *p* < 0.01, *** *p* < 0.001.

PiecewiseSEM results indicated that the elevation had a direct negative correlation with the biomass of *G. turkestanorum*, and also affected biomass indirectly through the effects on soil factors and functional traits. The direct effect (0.67) was substantially more pronounced than the indirect effects (0.46 and 0.07) (Figure 9).

## 4. Discussion

### 4.1. Different Organ Biomass Allocation Strategies of G. turkestanorum Along the Elevation Gradient

Our study found that the biomass of *G. turkestanorum* showed a decreasing trend with increasing elevation. This trend was primarily attributed to high soil water content (26.46%), which restricts plant growth rates and respiratory and metabolic activities, leading to a reduced number of *G. turkestanorum* in high elevation areas and consequently decreased biomass. This finding aligns with the previous research [23]. Additionally, temperature decrease is a key driver of changes in biomass allocation patterns. Studies on biomass allocation strategies of understory vegetation in China have shown that plants tend to prioritize below-ground biomass allocation under conditions of high elevation and low temperature [24]. Similar conclusions have been reached in studies of grassland ecosystems [25]. Consistent with these findings, our results revealed that the allometric growth trajectory of *G. turkestanorum* biomass varied with elevation; specifically, allocation to stems, leaves, and flowers decreased, while root allocation increased, mirroring the response of most plants to low temperatures in high elevation areas [26]. The phenomenon of low temperature at high elevation will not only cause damage to the above-ground part of plants, but also reduce the cumulative growth degree days, so the plants will adjust their biomass allocation strategy and increase the allocation of biomass to the underground part.

The distribution of plant biomass among organs is not only driven by environmental conditions, but also by genetic factors [27]. Our study found that *G. turkestanorum* allocated more biomass to flowers, with flower biomass accounting for 59.24% of the total biomass (Table S1), which is the natural result of its own long-term evolution. In the growing environment of *G. turkestanorum*, flowers need to be more conspicuous to attract limited pollinators, especially in high elevation areas where there may be fewer pollinators, so flower salience is critical for successful plant reproduction [28]. Additionally, with increasing elevation, *G. turkestanorum* allocated more biomass to leaves in the stem–leaf relationship (Figure 3E, Table 1). Initially, in the stem–flower relationship, *G. turkestanorum* allocated more biomass to flowers, but at high elevations, it shifted this allocation to stems (Figure 3F, Table 1). Regarding the leaf–flower relationship, *G. turkestanorum* decreased biomass allocation to flowers but increased it to leaves as elevation rose (Figure 3G, Table 1), which also proved that the biomass allocation of *G. turkestanorum* conforms to the hypothesis of optimal allocation theory. The low elevation environment in the study area was suitable for the growth of *G. turkestanorum*, and the plant nutrients were mainly used for the rapid growth and reproduction of the plants, so the biomass was higher. However, plants at high elevations adopt more conservative strategies to ensure their survival [29]. At high elevations, *G. turkestanorum* reduces investment in flowers and stems and increases it in leaves, indicating a shift towards prioritizing survival over reproduction and growth in response to harsh environments. Increasing leaf investment can enhance photosynthetic capacity, thereby meeting their survival needs [30].

### 4.2. Variations in Functional Traits of G. turkestanorum Along the Elevation Gradient

To cope with environmental changes, plants adjust their physiological and ecological functional traits. Therefore, functional traits not only represent the main strategies for plants to cope with the changing environment but also provide important information for assessing their ecosystems [17]. Our study showed that the chlorophyll content and leaf thickness were positively correlated with elevation, consistent with previous studies [31,32]. To adapt to the low-temperature environment at high elevations, plants increase leaf thickness to reduce heat loss and maintain internal leaf temperature. Additionally, in high-elevation areas, where ultraviolet radiation is enhanced, photosynthesis efficiency can be improved by increasing leaf thickness and mesophyll tissue, as well as chlorophyll content, to make full use of limited light resources—a survival strategy for *G. turkestanorum* to adapt to the environment [33]. Moreover, in this study, it was found that the leaf dry matter content and flower number correlated significantly and negatively with elevation, respectively. The relationship between leaf dry matter content and elevation was inconsistent with most research results [34,35]. However, our results are in agreement with those of studies on the functional traits of *Rhododendron aganniphum* leaves and the reproductive acclimatization of *Gentiana officinalis* at different elevations [36,37]. Leaf dry matter content reflects a plant’s nutrient production and storage capacity. In this study area, soil water content at a high elevation was higher (26.46%) than that at a low elevation (14.34%), allowing *G. turkestanorum* to obtain water more easily. Consequently, *G. turkestanorum* does not need to accumulate a large amount of dry matter in leaves for drought resistance 6. Due to the need for light, *G. turkestanorum* develops larger leaves for photosynthesis at high elevations (Figure 4), which may lead to a relative decrease in leaf dry matter content as the increase in leaf area exceeds the accumulation rate of dry matter. The significant decrease in flower numbers with increasing elevation may be due to the decrease in temperature affecting the survival of *G. turkestanorum*; alternatively, it may be due to the reduced pollinator availability in the low-temperature environment at high elevations, leading to a corresponding reduction in flower investment [36].

In summary, the complementary mechanisms among functional traits are associated with the self-adaptation and self-protection strategies of *G. turkestanorum* in its long-term survival environment. Under high-elevation environments, *G. turkestanorum* exhibits higher leaf thickness and lower leaf dry matter content, plant dwarfness (Figure 4), and low resource acquisition ability, all of which contribute to slow plant growth and a focus on survival. This is characteristic of a ‘conservative’ survival strategy. Conversely, low-elevation environments are conducive to the rapid growth of *G. turkestanorum*, and adopting a ‘pioneering’ survival strategy allows it to achieve higher growth rates and resource acquisition abilities, fulfilling its survival and reproductive needs.

### 4.3. The Driving of Environmental Factors and Functional Traits

The results of SMA, variance decomposition, and piecewiseSEM showed that the effect of soil factors on the biomass of *G. turkestanorum* exhibited a higher explanatory power compared to functional traits. Our study found that soil water content and pH had significant effects on the biomass of *G. turkestanorum*, and soil water content had a negative correlation with biomass, which was consistent with previous studies [23]. Water is necessary for plant growth, but excessive water may lead to water stress. On the one hand, air in the soil is crowded out, resulting in root hypoxia that inhibits plant respiration; on the other hand, leaf stomata are closed, reducing CO_2_ entry and photosynthetic efficiency, and ultimately affecting plant growth and biomass accumulation [38]. In addition, available P, total N, and ammonium N showed significant negative correlations with the biomass of *G. turkestanorum*, which was inconsistent with previous studies [39]. The reason may be that water stress at a high elevation inhibited root growth and showed a lower root length and diameter, which would directly affect the uptake of available P and N by plants in soil. This may also be the reason why soil water content can significantly and accurately predict the biomass of *G. turkestanorum*. In our study, we also found that flower number, crown width, root length, root diameter, and leaf dry matter content also had significant positive effects on the biomass of *G. turkestanorum*, which was consistent with previous findings [16,40,41]. Notably, flower number and root diameter significantly influenced the accurate prediction of *G. turkestanorum* biomass, which may be because flower biomass accounted for 59.24% of the total biomass, and flowers were reproductive organs, and the increase in their number reflected the transition from vegetative to reproductive growth of plants. This transition is also accompanied by an increase in biomass [42]. Plants with larger crown widths are exposed to more light and have a greater ability to obtain resources. Through photosynthesis, light energy is converted into chemical energy and stored in organic matter, which is conducive to the accumulation of biomass. Root length and root diameter represent the ability of plants to directly obtain nutrients, and the larger they are, the larger the surface area of roots in contact with soil, thus enhancing the ability of roots to explore and absorb nutrients, and contributing to the accumulation of biomass [17]. Surprisingly, the biomass of *G. turkestanorum* does not significantly correlate with leaf area, specific leaf area, and leaf thickness, as other studies have shown [43], which may be caused by the plasticity of leaf traits, which can be adjusted according to environmental changes. This plasticity reduced the direct correlation between leaf traits and biomass [44], but indirectly affected the biomass of *G. turkestanorum* through various mechanisms, while the dry matter of leaves contained carbohydrates such as starch and nutrients such as nitrogen, phosphorus, and potassium [45]. Energy supply meets plant growth needs and therefore has a significant positive correlation with biomass. The piecewiseSEM results indicated that increased elevation directly affected the biomass of *G. turkestanorum* and also had indirect effects through other pathways. The direct effect was more pronounced, as it directly altered the growth environmental conditions for *G. turkestanorum*, including temperature and soil moisture content—key factors influencing biomass accumulation. Although indirect factors were also important, they did not significantly affect the biomass accumulation of *G. turkestanorum*. These indirect factors are typically controlled and regulated by elevation [46].

## 5. Conclusions

The biomass of *G. turkestanorum* was primarily composed of flowers, accounting for 59.24% of the total biomass, and followed the biomass allocation strategy of the optimal allocation theory across a range of elevations. At lower elevations, a ‘pioneering’ biomass allocation type was dominant, while a ‘conservative’ type was observed at higher elevations. The functional traits of leaf and flower of *G. turkestanorum* were significantly affected by elevation. Elevation, soil moisture content, and functional traits of flower and root were closely related to the biomass of *G. turkestanorum*. Elevation directly influenced the biomass of *G. turkestanorum* and also had indirect effects through other pathways; however, the direct effect was larger than the indirect effect. The results of this study not only enrich the understanding of the growth strategy of *G. turkestanorum* in elevation gradient, but also provide theoretical basis for the protection, management, and sustainable use of medicinal plants in grassland.

## Figures and Tables

**Table 1:** Determination of allometric trajectory slope and intercept of the biomass of *G. turkestanorum* under different elevations gradients.

		Allometric Slope			Allometric Intercept			p (H0: Slope Are Equal)	p (H0: Intercept Are Equal)
y	x	Low	Middle	High	Low	Middle	High		
AGB	BGB	1.820	1.084	0.864	1.151	1.194	1.039	<0.001	<0.001
SB	RB	1.943	0.729	1.330	0.476	0.541	0.672	<0.001	>0.05
LB	RB	0.926	1.003	1.629	0.404	0.266	0.476	<0.001	>0.05
FB	RB	2.273	1.289	0.559	0.876	1.008	0.651	<0.001	<0.001
LB	SB	0.477	1.375	1.225	0.177	−0.479	−0.347	<0.001	<0.001
FB	SB	1.170	1.767	0.420	0.320	0.051	0.368	<0.001	>0.05
FB	LB	2.454	1.286	0.343	−0.115	0.667	0.487	<0.001	<0.001

**Table 2:** Significance test of factors affecting the biomass of *G. turkestanorum*.

Elevation-Soil Factors				Functional Traits			
Impact Factors	Axis I Score	Axis II Score	R^2^	Impact Factors	Axis I Score	Axis II Score	R^2^
SW	0.828	−0.354	0.625 ***	NOF	−0.752	−0.250	0.335 ***
Elevation	0.789	−0.261	0.587 ***	RD	−0.807	0.313	0.313 **
pH	−0.573	−0.560	0.213 *	CD	−0.724	0.395	0.256 **
AP	0.479	0.198	0.152 *	RL	−0.605	0.019	0.232 **
TN	0.382	0.131	0.148 *	LDMC	−0.612	0.368	0.127 *
AN	0.338	0.085	0.144 *	LT	0.366	0.453	0.056

* *p* < 0.05, ** *p* < 0.01, *** *p* < 0.001.

## Data Availability

Data are contained within the article.

## Supplementary Materials

The following supporting information can be downloaded at: https://www.mdpi.com/article/10.3390/plants13243463/s1, Table S1: distribution characteristics of root, stem, leaf, flower, total biomass, and BGB/AGB at elevation; Table S2: elevation distribution of functional Traits of *G. turkestanorum*; Table S3: significance test of factors affecting the biomass of *G. turkestanorum*.
